# Methyl ({[(4*E*)-1,3-dimethyl-2,6-di­phenyl­piperidin-4-yl­idene]amino}­oxy)acetate

**DOI:** 10.1107/S1600536814006667

**Published:** 2014-04-02

**Authors:** T. Mohandas, M. Velayutham Pillai, T. Vidhyasagar, A. Pasupathy, P. Sakthivel

**Affiliations:** a Department of Physics, Shri Angalamman College of Engineering and Technology, Siruganoor, Tiruchirappalli, India; b Department of Chemistry, Annamalai University, Annamalainagar, Chidambaram, India; c Department of Chemistry, Urumu Dhanalakshmi College, Tiruchirappalli 620 019, India; d Department of Physics, Urumu Dhanalakshmi College, Tiruchirappalli 620 019, India

## Abstract

In the title compound, C_22_H_26_N_2_O_3_, the piperidine ring exhibits a chair conformation. The phenyl rings attached to the piperidine at the 2- and 6-positions have axial orientations. These rings make a dihedral angle of 49.75 (11)°. The amino­oxy acetate group attached at the 4-position has an equatorial orientation. In the crystal, inversion dimers linked by pairs of C—H⋯π inter­actions occur.

## Related literature   

For background and the importance of oxime ethers, see: Crichlow *et al.* (2007 ▶). For a study of the *in vitro* anti­proliferative activity of oxyme ether derivatives, see: Parthiban *et al.* (2011 ▶). For their effects on the senescence of cut carnation flowers, see: Zeng *et al.* (2012 ▶). For ring conformations, see: Cremer & Pople (1975 ▶). For related structures, see: Park *et al.* (2012*a* ▶,*b* ▶).
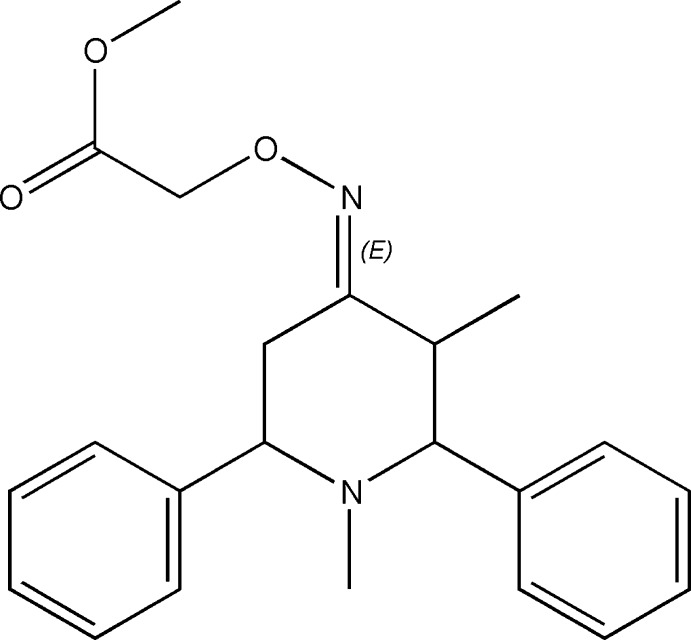


## Experimental   

### 

#### Crystal data  

C_22_H_26_N_2_O_3_
*M* *_r_* = 366.45
Monoclinic, 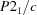

*a* = 8.1662 (9) Å
*b* = 15.0229 (16) Å
*c* = 16.2889 (19) Å
β = 93.903 (6)°
*V* = 1993.7 (4) Å^3^
*Z* = 4
Mo *K*α radiation
μ = 0.08 mm^−1^
*T* = 295 K
0.35 × 0.35 × 0.30 mm

#### Data collection  

Bruker Kappa APEXII CCD diffractometer
Absorption correction: multi-scan (*SADABS*; Bruker, 2008 ▶) *T*_min_ = 0.972, *T*_max_ = 0.976
17582 measured reflections
17582 independent reflections
12046 reflections with *I* > 2σ(*I*)

#### Refinement  

*R*[*F* ^2^ > 2σ(*F* ^2^)] = 0.071
*wR*(*F* ^2^) = 0.214
*S* = 1.06
17582 reflections
249 parameters
H-atom parameters constrained
Δρ_max_ = 0.31 e Å^−3^
Δρ_min_ = −0.29 e Å^−3^

### 

Data collection: *APEX2* (Bruker, 2008 ▶); cell refinement: *APEX2* and *SAINT* (Bruker, 2008 ▶); data reduction: *SAINT*; program(s) used to solve structure: *SHELXS97* (Sheldrick, 2008 ▶); program(s) used to refine structure: *SHELXL97* (Sheldrick, 2008 ▶); molecular graphics: *ORTEP-3 for Windows* (Farrugia, 2012 ▶); software used to prepare material for publication: *PLATON* (Spek, 2009 ▶).

## Supplementary Material

Crystal structure: contains datablock(s) global, I. DOI: 10.1107/S1600536814006667/rk2422sup1.cif

Structure factors: contains datablock(s) I. DOI: 10.1107/S1600536814006667/rk2422Isup2.hkl

Supporting information file. DOI: 10.1107/S1600536814006667/rk2422Isup3.cml

CCDC reference: 993682

Additional supporting information:  crystallographic information; 3D view; checkCIF report

## Figures and Tables

**Table 1:** Hydrogen-bond geometry (Å, °) *Cg*2 is the centroid of the C1–C6 ring.

*D*—H⋯*A*	*D*—H	H⋯*A*	*D*⋯*A*	*D*—H⋯*A*
C22—H22*B*⋯*Cg*2^i^	0.96	2.76	3.525 (3)	138.00

Symmetry code: (i) 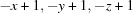.

**Fig. 1.**
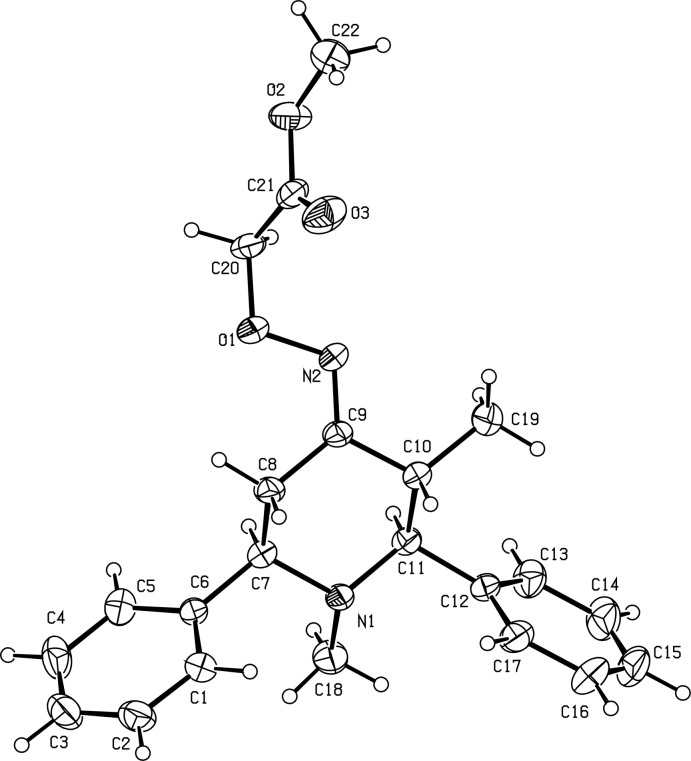
The molecular structure and labelling scheme for title compound. Displacement ellipsoids are drawn at the 50% probability level. H atoms are presented as a small spheres of arbitrary radius.

**Fig. 2.**
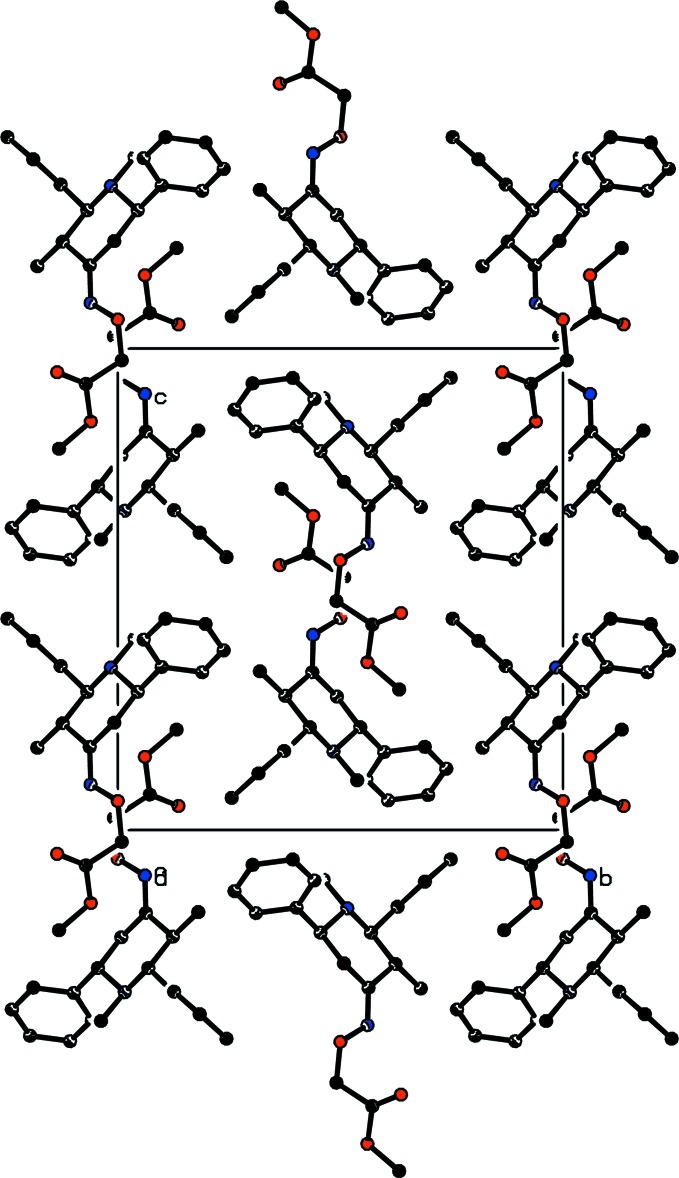
A packing diagram for title compound.

## supplementary crystallographic information

### 1. Comment

Oxime ethers are serving as important synthetic intermediate and have been employed as starting materials for both synthetic and medicinal chemistry (Crichlow *et al.*, 2007).

Oxime ether derivatives of 2,6-diphenyl-piperidine–4-one with *N*-methyl substituent enhanced the efficacy of the *in vitro* antiproliferative activity against human cervical carcinoma cell lines (Parthiban *et al.*, 2011). Recently oxime ether derivatives of aminooxy acetic acid were synthesized and studied for their effects on the senescence of cut carnation flowers (Zeng *et al.*, 2012). Due to the above importance, the crystal data for the title compound was carried out by X–ray diffraction.

Piperidine ring (C7–C11/N1) exists in a chair conformation with the puckering parameter Q = 0.5566Å, θ = 1.70 (19)°, φ= 23 (7)° (*syn periplanar*).

The bond distances and bond angles in the title compound agree very well with the corresponding values reported in closely related compound (Park *et al.*, 2012*a*; 2012*b*).

The main plane of piperidine ring makes the dihedral angle of 81.82° and 86.73° with the phenyl rings at the 2 and 6 positions respectively. The dihedral angle between the two phenyl rings is 49.75°. The crystal is stabilized by C—H···O interactions. The packing is further stabilized by C—H···π and C22—H22B···Cg2^i^ interactions, where *Cg*2 is the centroid of (C1–C6) ring. Symmetry code: (i) 1*-x*, 1*-y*, 1*-z*.

### 2. Experimental

To a stirred mixture of 1,3-dimethyl-2,6-diphenylpiperidin-4-one oxime (0.88 g, 3 mmol) and K_2_CO_3_ (0.42 g, 1 eq.) in acetonitrile (15 ml) at 353 K, methyl chloroacetate (0.25 ml, 1 eq) was added dropwise over a period of 5 min and stirring continued for 7.5 hrs at the same condition. The progress of the reaction was monitored by *TLC*. After completion of the reaction K_2_CO_3_ was removed by filtration and the solvent was evaporated to getcrude product. Pure product was obtained by coloumn chromotography using petroleum ether/ethyl acetate (9.5/0.5) mixture as the eluent. Yield: 0.77 g (70%).

### 3. Refinement

All the hydrogen atoms were geometrically fixed and allowed to ride on their parent atoms with C—H = 0.93–0.98 Å and *U*_iso_(H) = 1.5*U*_eq_(C) for methyl H atoms and *U*_iso_(H) = 1.2*U*_eq_(C) for other H.

### Crystal data

**Table:**

C_22_H_26_N_2_O_3_	*F*(000) = 784
*M**_r_* = 366.45	*D*_x_ = 1.221 Mg m^−^^3^
Monoclinic, *P*2_1_/*c*	Mo *K*α radiation, λ = 0.71073 Å
Hall symbol: -P 2ybc	Cell parameters from 17582 reflections
*a* = 8.1662 (9) Å	θ = 1.9–25.7°
*b* = 15.0229 (16) Å	µ = 0.08 mm^−^^1^
*c* = 16.2889 (19) Å	*T* = 295 K
β = 93.903 (6)°	Block, colourless
*V* = 1993.7 (4) Å^3^	0.35 × 0.35 × 0.30 mm
*Z* = 4	

### Data collection

**Table:**

Bruker Kappa APEXII CCD diffractometer	17582 independent reflections
Radiation source: fine–focus sealed tube	12046 reflections with *I* > 2σ(*I*)
Graphite monochromator	*R*_int_ = 0.000
Detector resolution: 18.4 pixels mm^-1^	θ_max_ = 25.7°, θ_min_ = 1.9°
ω and φ scans	*h* = −9→9
Absorption correction: multi-scan (*SADABS*; Bruker, 2008)	*k* = −18→18
*T*_min_ = 0.972, *T*_max_ = 0.976	*l* = −19→16
17582 measured reflections	

### Refinement

**Table:**

Refinement on *F*^2^	Secondary atom site location: difference Fourier map
Least-squares matrix: full	Hydrogen site location: inferred from neighbouring sites
*R*[*F*^2^ > 2σ(*F*^2^)] = 0.071	H-atom parameters constrained
*wR*(*F*^2^) = 0.214	*w* = 1/[σ^2^(*F*_o_^2^) + (0.0418*P*)^2^ + 3.4429*P*] where *P* = (*F*_o_^2^ + 2*F*_c_^2^)/3
*S* = 1.06	(Δ/σ)_max_ < 0.001
17582 reflections	Δρ_max_ = 0.31 e Å^−^^3^
249 parameters	Δρ_min_ = −0.29 e Å^−^^3^
0 restraints	Extinction correction: *SHELXL97* (Sheldrick, 2008), Fc^*^=kFc[1+0.001xFc^2^λ^3^/sin(2θ)]^-1/4^
Primary atom site location: structure-invariant direct methods	Extinction coefficient: 0.0042 (7)

### Special details

**Table:**

Geometry. Bond distances, angles *etc*. have been calculated using the rounded fractional coordinates. All s.u.'s are estimated from the variances of the (full) variance–covariance matrix. The cell s.u.'s are taken into account in the estimation of distances, angles and torsion angles.
Refinement. Refinement of *F*^2^ against ALL reflections. The weighted *R*–factor *wR* and goodness of fit *S* are based on *F*^2^, conventional *R*–factors *R* are based on *F*, with *F* set to zero for negative *F*^2^. The threshold expression of *F*^2^ > σ(*F*^2^) is used only for calculating *R*–factors(gt) *etc*. and is not relevant to the choice of reflections for refinement. *R*–factors based on *F*^2^ are statistically about twice as large as those based on *F*, and *R*–factors based on ALL data will be even larger.

### Fractional atomic coordinates and isotropic or equivalent isotropic displacement parameters (Å^2^)

**Table:**

	*x*	*y*	*z*	*U*_iso_*/*U*_eq_	
O1	0.68977 (19)	0.49995 (10)	0.43999 (8)	0.0606 (6)	
O2	0.7546 (2)	0.43922 (10)	0.65189 (9)	0.0649 (6)	
O3	0.6248 (2)	0.36381 (11)	0.54968 (10)	0.0771 (7)	
N1	0.83698 (18)	0.48449 (10)	0.16289 (9)	0.0383 (5)	
N2	0.7999 (2)	0.43901 (11)	0.40538 (10)	0.0467 (6)	
C1	0.5090 (3)	0.55836 (14)	0.10496 (12)	0.0499 (7)	
C2	0.3910 (3)	0.60714 (17)	0.06143 (13)	0.0619 (9)	
C3	0.3809 (3)	0.69704 (19)	0.07252 (15)	0.0684 (10)	
C4	0.4887 (3)	0.73784 (16)	0.12720 (17)	0.0680 (10)	
C5	0.6074 (3)	0.68953 (14)	0.17255 (14)	0.0529 (8)	
C6	0.6190 (2)	0.59865 (12)	0.16163 (11)	0.0381 (7)	
C7	0.7430 (2)	0.54460 (12)	0.21323 (11)	0.0394 (7)	
C8	0.6541 (2)	0.49215 (14)	0.27705 (11)	0.0459 (7)	
C9	0.7736 (2)	0.43693 (13)	0.32754 (11)	0.0390 (7)	
C10	0.8732 (2)	0.37623 (12)	0.27849 (11)	0.0401 (7)	
C11	0.9567 (2)	0.43131 (12)	0.21322 (11)	0.0387 (6)	
C12	1.0538 (3)	0.37151 (13)	0.15962 (12)	0.0427 (7)	
C13	1.2220 (3)	0.37356 (15)	0.16396 (14)	0.0583 (9)	
C14	1.3111 (3)	0.31608 (18)	0.11732 (17)	0.0757 (11)	
C15	1.2312 (4)	0.25588 (19)	0.06656 (17)	0.0820 (11)	
C16	1.0624 (4)	0.25213 (18)	0.06119 (15)	0.0792 (11)	
C17	0.9742 (3)	0.30997 (15)	0.10737 (13)	0.0575 (8)	
C18	0.9212 (3)	0.53774 (15)	0.10310 (13)	0.0606 (9)	
C19	0.9942 (3)	0.31926 (14)	0.32933 (13)	0.0579 (8)	
C20	0.7357 (3)	0.50883 (14)	0.52479 (11)	0.0542 (8)	
C21	0.6970 (3)	0.42816 (15)	0.57362 (13)	0.0490 (8)	
C22	0.7303 (3)	0.36794 (16)	0.70832 (15)	0.0774 (10)	
H1	0.51517	0.49729	0.09631	0.0598*	
H2	0.31720	0.57886	0.02404	0.0743*	
H3	0.30077	0.72999	0.04279	0.0822*	
H4	0.48279	0.79915	0.13434	0.0816*	
H5	0.67945	0.71831	0.21045	0.0635*	
H7	0.81993	0.58569	0.24234	0.0473*	
H8A	0.59930	0.53296	0.31232	0.0551*	
H8B	0.57149	0.45413	0.24952	0.0551*	
H10	0.79627	0.33561	0.24872	0.0481*	
H11	1.03388	0.47245	0.24214	0.0465*	
H13	1.27740	0.41420	0.19880	0.0700*	
H14	1.42514	0.31870	0.12074	0.0909*	
H15	1.29084	0.21721	0.03544	0.0981*	
H16	1.00786	0.21087	0.02665	0.0952*	
H17	0.86013	0.30759	0.10335	0.0690*	
H18A	0.99136	0.58048	0.13174	0.0910*	
H18B	0.84144	0.56824	0.06736	0.0910*	
H18C	0.98595	0.49935	0.07109	0.0910*	
H19A	1.08019	0.35618	0.35384	0.0869*	
H19B	1.04053	0.27561	0.29462	0.0869*	
H19C	0.93886	0.28981	0.37181	0.0869*	
H20A	0.67918	0.55973	0.54619	0.0650*	
H20B	0.85263	0.52040	0.53176	0.0650*	
H22A	0.64140	0.33102	0.68696	0.1162*	
H22B	0.70458	0.39208	0.76047	0.1162*	
H22C	0.82870	0.33301	0.71532	0.1162*	

### Atomic displacement parameters (Å^2^)

**Table:**

	*U*^11^	*U*^22^	*U*^33^	*U*^12^	*U*^13^	*U*^23^
O1	0.0757 (11)	0.0737 (11)	0.0332 (8)	0.0271 (9)	0.0097 (7)	−0.0022 (7)
O2	0.0901 (12)	0.0665 (10)	0.0372 (9)	−0.0126 (9)	−0.0029 (8)	0.0039 (8)
O3	0.1051 (14)	0.0639 (11)	0.0629 (11)	−0.0216 (10)	0.0111 (10)	−0.0182 (9)
N1	0.0392 (9)	0.0444 (9)	0.0321 (9)	0.0053 (7)	0.0094 (8)	0.0040 (7)
N2	0.0561 (11)	0.0505 (10)	0.0343 (10)	0.0116 (9)	0.0086 (8)	−0.0057 (8)
C1	0.0560 (13)	0.0519 (13)	0.0414 (12)	0.0069 (11)	0.0010 (11)	0.0031 (10)
C2	0.0577 (14)	0.0802 (18)	0.0469 (14)	0.0085 (13)	−0.0040 (12)	0.0074 (13)
C3	0.0623 (16)	0.081 (2)	0.0624 (17)	0.0243 (14)	0.0081 (14)	0.0244 (14)
C4	0.0731 (17)	0.0438 (14)	0.090 (2)	0.0148 (13)	0.0279 (15)	0.0214 (14)
C5	0.0514 (13)	0.0458 (13)	0.0627 (15)	0.0006 (11)	0.0123 (12)	−0.0008 (11)
C6	0.0406 (11)	0.0401 (12)	0.0344 (11)	0.0043 (9)	0.0078 (9)	0.0030 (9)
C7	0.0425 (12)	0.0414 (11)	0.0346 (11)	0.0001 (9)	0.0043 (9)	−0.0041 (9)
C8	0.0427 (12)	0.0629 (14)	0.0328 (11)	0.0124 (10)	0.0071 (10)	0.0051 (10)
C9	0.0405 (11)	0.0458 (12)	0.0315 (11)	0.0015 (9)	0.0091 (9)	0.0032 (9)
C10	0.0438 (11)	0.0429 (12)	0.0342 (11)	0.0044 (9)	0.0065 (9)	−0.0005 (9)
C11	0.0350 (10)	0.0447 (11)	0.0365 (11)	−0.0002 (9)	0.0028 (9)	−0.0046 (9)
C12	0.0478 (13)	0.0468 (12)	0.0349 (11)	0.0055 (10)	0.0130 (10)	0.0030 (9)
C13	0.0483 (14)	0.0612 (15)	0.0672 (16)	0.0038 (11)	0.0174 (12)	0.0001 (12)
C14	0.0597 (16)	0.0826 (19)	0.088 (2)	0.0216 (14)	0.0294 (15)	0.0069 (16)
C15	0.099 (2)	0.079 (2)	0.0726 (19)	0.0311 (17)	0.0398 (17)	−0.0027 (16)
C16	0.101 (2)	0.0803 (19)	0.0580 (16)	0.0145 (16)	0.0181 (16)	−0.0243 (14)
C17	0.0589 (14)	0.0685 (16)	0.0455 (13)	0.0077 (12)	0.0061 (12)	−0.0092 (12)
C18	0.0643 (15)	0.0670 (16)	0.0537 (14)	0.0106 (12)	0.0265 (12)	0.0184 (12)
C19	0.0609 (14)	0.0614 (15)	0.0524 (14)	0.0172 (12)	0.0105 (12)	0.0028 (11)
C20	0.0788 (16)	0.0557 (14)	0.0283 (11)	0.0030 (12)	0.0058 (11)	−0.0052 (10)
C21	0.0581 (13)	0.0516 (14)	0.0383 (12)	0.0028 (12)	0.0107 (11)	−0.0102 (10)
C22	0.105 (2)	0.0694 (17)	0.0589 (16)	0.0111 (15)	0.0129 (15)	0.0156 (13)

### Geometric parameters (Å, º)

**Table:**

O1—N2	1.426 (2)	C20—C21	1.495 (3)
O1—C20	1.413 (2)	C1—H1	0.9300
O2—C21	1.339 (3)	C2—H2	0.9300
O2—C22	1.434 (3)	C3—H3	0.9300
O3—C21	1.185 (3)	C4—H4	0.9300
N1—C7	1.471 (2)	C5—H5	0.9300
N1—C11	1.469 (2)	C7—H7	0.9800
N1—C18	1.467 (3)	C8—H8A	0.9700
N2—C9	1.272 (2)	C8—H8B	0.9700
C1—C2	1.369 (3)	C10—H10	0.9800
C1—C6	1.383 (3)	C11—H11	0.9800
C2—C3	1.366 (4)	C13—H13	0.9300
C3—C4	1.355 (4)	C14—H14	0.9300
C4—C5	1.384 (3)	C15—H15	0.9300
C5—C6	1.381 (3)	C16—H16	0.9300
C6—C7	1.508 (2)	C17—H17	0.9300
C7—C8	1.527 (3)	C18—H18A	0.9600
C8—C9	1.486 (3)	C18—H18B	0.9600
C9—C10	1.490 (3)	C18—H18C	0.9600
C10—C11	1.542 (2)	C19—H19A	0.9600
C10—C19	1.511 (3)	C19—H19B	0.9600
C11—C12	1.514 (3)	C19—H19C	0.9600
C12—C13	1.371 (3)	C20—H20A	0.9700
C12—C17	1.388 (3)	C20—H20B	0.9700
C13—C14	1.388 (4)	C22—H22A	0.9600
C14—C15	1.362 (4)	C22—H22B	0.9600
C15—C16	1.376 (5)	C22—H22C	0.9600
C16—C17	1.383 (4)		
			
N2—O1—C20	108.33 (15)	C6—C5—H5	120.00
C21—O2—C22	117.57 (18)	N1—C7—H7	108.00
C7—N1—C11	112.06 (14)	C6—C7—H7	108.00
C7—N1—C18	108.74 (15)	C8—C7—H7	108.00
C11—N1—C18	110.05 (15)	C7—C8—H8A	110.00
O1—N2—C9	109.98 (15)	C7—C8—H8B	110.00
C2—C1—C6	120.9 (2)	C9—C8—H8A	110.00
C1—C2—C3	120.5 (2)	C9—C8—H8B	110.00
C2—C3—C4	119.5 (2)	H8A—C8—H8B	108.00
C3—C4—C5	120.9 (2)	C9—C10—H10	107.00
C4—C5—C6	120.1 (2)	C11—C10—H10	107.00
C1—C6—C5	118.14 (18)	C19—C10—H10	107.00
C1—C6—C7	121.15 (17)	N1—C11—H11	108.00
C5—C6—C7	120.65 (17)	C10—C11—H11	108.00
N1—C7—C6	112.08 (14)	C12—C11—H11	108.00
N1—C7—C8	110.68 (15)	C12—C13—H13	119.00
C6—C7—C8	108.96 (14)	C14—C13—H13	119.00
C7—C8—C9	109.89 (14)	C13—C14—H14	120.00
N2—C9—C8	126.73 (17)	C15—C14—H14	120.00
N2—C9—C10	119.23 (16)	C14—C15—H15	120.00
C8—C9—C10	114.01 (15)	C16—C15—H15	120.00
C9—C10—C11	108.93 (15)	C15—C16—H16	120.00
C9—C10—C19	114.41 (16)	C17—C16—H16	120.00
C11—C10—C19	112.23 (15)	C12—C17—H17	120.00
N1—C11—C10	111.59 (13)	C16—C17—H17	120.00
N1—C11—C12	110.84 (15)	N1—C18—H18A	109.00
C10—C11—C12	110.65 (15)	N1—C18—H18B	109.00
C11—C12—C13	121.28 (18)	N1—C18—H18C	109.00
C11—C12—C17	120.5 (2)	H18A—C18—H18B	109.00
C13—C12—C17	118.2 (2)	H18A—C18—H18C	109.00
C12—C13—C14	121.2 (2)	H18B—C18—H18C	109.00
C13—C14—C15	119.9 (2)	C10—C19—H19A	109.00
C14—C15—C16	120.2 (3)	C10—C19—H19B	109.00
C15—C16—C17	119.7 (2)	C10—C19—H19C	109.00
C12—C17—C16	120.8 (2)	H19A—C19—H19B	109.00
O1—C20—C21	113.08 (17)	H19A—C19—H19C	109.00
O2—C21—O3	123.3 (2)	H19B—C19—H19C	109.00
O2—C21—C20	109.43 (18)	O1—C20—H20A	109.00
O3—C21—C20	127.2 (2)	O1—C20—H20B	109.00
C2—C1—H1	120.00	C21—C20—H20A	109.00
C6—C1—H1	120.00	C21—C20—H20B	109.00
C1—C2—H2	120.00	H20A—C20—H20B	108.00
C3—C2—H2	120.00	O2—C22—H22A	109.00
C2—C3—H3	120.00	O2—C22—H22B	109.00
C4—C3—H3	120.00	O2—C22—H22C	109.00
C3—C4—H4	120.00	H22A—C22—H22B	109.00
C5—C4—H4	120.00	H22A—C22—H22C	109.00
C4—C5—H5	120.00	H22B—C22—H22C	109.00
			
C20—O1—N2—C9	−172.81 (17)	C6—C7—C8—C9	178.85 (15)
N2—O1—C20—C21	−71.9 (2)	N1—C7—C8—C9	55.18 (19)
C22—O2—C21—C20	−179.44 (19)	C7—C8—C9—C10	−55.2 (2)
C22—O2—C21—O3	1.7 (3)	C7—C8—C9—N2	122.7 (2)
C18—N1—C11—C12	−57.7 (2)	N2—C9—C10—C11	−124.12 (18)
C7—N1—C11—C12	−178.86 (15)	N2—C9—C10—C19	2.4 (2)
C11—N1—C7—C6	−179.50 (14)	C8—C9—C10—C11	53.95 (19)
C18—N1—C7—C6	58.62 (19)	C8—C9—C10—C19	−179.55 (16)
C11—N1—C7—C8	−57.64 (18)	C19—C10—C11—C12	54.6 (2)
C18—N1—C11—C10	178.47 (15)	C9—C10—C11—C12	−177.68 (15)
C18—N1—C7—C8	−179.52 (15)	C19—C10—C11—N1	178.49 (15)
C7—N1—C11—C10	57.34 (18)	C9—C10—C11—N1	−53.77 (18)
O1—N2—C9—C8	3.3 (3)	C10—C11—C12—C17	66.3 (2)
O1—N2—C9—C10	−178.95 (15)	C10—C11—C12—C13	−110.6 (2)
C2—C1—C6—C7	176.48 (19)	N1—C11—C12—C17	−58.0 (2)
C6—C1—C2—C3	0.8 (3)	N1—C11—C12—C13	125.0 (2)
C2—C1—C6—C5	−0.6 (3)	C11—C12—C13—C14	177.3 (2)
C1—C2—C3—C4	−0.1 (4)	C13—C12—C17—C16	0.2 (3)
C2—C3—C4—C5	−0.8 (4)	C11—C12—C17—C16	−176.8 (2)
C3—C4—C5—C6	1.0 (4)	C17—C12—C13—C14	0.2 (3)
C4—C5—C6—C1	−0.3 (3)	C12—C13—C14—C15	−0.5 (4)
C4—C5—C6—C7	−177.4 (2)	C13—C14—C15—C16	0.2 (4)
C1—C6—C7—N1	51.3 (2)	C14—C15—C16—C17	0.2 (4)
C5—C6—C7—C8	105.5 (2)	C15—C16—C17—C12	−0.4 (4)
C5—C6—C7—N1	−131.70 (19)	O1—C20—C21—O3	−5.6 (4)
C1—C6—C7—C8	−71.5 (2)	O1—C20—C21—O2	175.58 (18)

### Hydrogen-bond geometry (Å, º)

*Cg*2 is the centroid of the C1–C6 ring.

**Table:**

*D*—H···*A*	*D*—H	H···*A*	*D*···*A*	*D*—H···*A*
C8—H8*A*···O1	0.97	2.22	2.653 (2)	106
C22—H22*A*···O3	0.96	2.28	2.668 (3)	103
C22—H22*B*···*Cg*2^i^	0.96	2.76	3.525 (3)	138

Symmetry code: (i) −*x*+1, −*y*+1, −*z*+1.
